# Toxicology

**Published:** 1837-07

**Authors:** 


### TOXICOLOGY.

*Experiments on the Effects of the Hydrated Per-Oxyde of Iron as an Antidote to Arsenious Acid.* By Dr. VON SPECZ, Professor of Chemistry in the Theresian Academy of Vienna.

IN the year 1834, as our readers are aware, Dr. R. W. Bunsen and Dr. A. A. Berthold published a small work at Göttingen, on the use of hydrated per-oxyde of iron as an antidote to arsenic, in which they claimed for this remedy all the properties of a true specific. With the view of testing the accuracy of their results, Dr. Von Specz prepared a large quantity of the hydrated peroxyde of iron, and instituted a series of experiments with it, on animals.

*Experiment* 1. On the 5th of October, 1834, at ten o'clock in the forenoon, twelve grains of white arsenic, mixed with a portion of boiled meat, was administered to a cat six months old. At twelve, retching and vomiting commenced. An attempt was made to administer the antidote mixed with water, by means of a tin funnel, but the animal was so extremely restive, that no more than a drachm of the hydrated peroxyde could be given. Death occurred about one o'clock.

*Experiment* 2. On the 15th of June, 1835, at ten o'clock in the forenoon, a scruple of the white oxyde of arsenic was given in some sausage to a dog six months old. Half an hour afterwards, Dr. Von Specz attempted to introduce the antidote, by means of a syringe furnished with an elastic tube. The dog, however, bit the tube, and was otherwise so unmanageable, that scarcely any could be administered. He died at half-past twelve.

Dr. Von Specz finding that the hydrated peroxyde of iron could not be given in a sufficient dose in this way, made the following change in the mode of exhibition.

*Experiment* 3. On the 28th of October, 1835, at ten o'clock in the forenoon, a drachm of finely powdered arsenic was administered to a mastiff six months old, which had been sparingly fed the day before. About five minutes afterwards, an ounce of the dry hydrated peroxide of iron, finely powdered, and mixed with a sufficient quantity of fryed liver pudding, was laid before him. He ate the whole, and afterwards drank about three ounces of milk. About eleven o'clock, he had some fluid evacuations from the bowels, but still appeared lively. About three o'clock P. M. he looked dejected, went to his bed, and lay there quietly; during the night he had five fluid evacuations. On the 29th, he looked dejected, did not stir from his bed, and neither ate nor drank. On the 30th he ate a little meat, drank about six ounces of milk, and returned to his bed. On the morning of the 1st of November, he was quite lively, and ate every thing that was offered to him.

*Experiment* 4. On the 10th of December, 1835, at ten o'clock in the forenoon, a drachm of finely powdered arsenic was given in some pudding to a bitch, twelve months old. Five minutes afterwards, an ounce of the antidote was given, mixed with liver pudding, the whole of which was devoured by the animal. At eleven o'clock she went to her bed, appeared shy and timid, and refused to eat. She remained quietly in her bed the whole night, without vomiting or purging. On the morning of the 11th she had three fluid evacuations from the bowels, and did not appear to be ill; on the 12th she was running about as usual.

The foregoing mode of exhibiting the antidote to animals may be employed until vomiting commences; after this occurrence, it must be administered by means of a syringe.

As one sixth of the quantity of arsenic administered in Experiments 3 and 4, is more than sufficient to kill a dog, Dr. Von Specz looks upon the hydrated peroxide of iron as a true specific, and thinks that its failure in any given case is to be attributed to its being employed too late, or given in too small a quantity. In order to ensure the giving of a sufficient dose, ten times the quantity of hydrated peroxide of iron must be administered; but a much larger quantity may be safely given, as this remedy does not exercise any deleterious effect on the animal œconomy. In order to obtain the remedy in a state of purity, and free from any admixture of copper, he recommends the precipitation of the iron by ammonia, and states that this preparation when properly made, and preserved in bottles with good glass stoppers, will retain its virtues for a very considerable time. The following is the mode of exhibition which he recommends in cases of poisoning by arsenic.—℞. Olei Amygdal. dulcis, Pulv. Gummi Arabici, Pulv. Sacchari Albi *aa* ʒij. tere simul et affunde, sensim terendo, Aquæ destillatæ ℥xv.; Hydratis Ferrici ℥iij.; of this mixture, previously well shaken, a dessert-spoonful is to be given every three minutes.

Dr. Von Specz resumed his investigations the following year, but instead of the pure hydrated peroxyde, he employed substances in which it is known to exist in considerable quantity, and which require no previous preparation, namely, rust of iron, and hæmatite (red iron ore.)

*Experiment* 1. On the 16th of January, 1836, at ten o'clock in the forenoon, a drachm of finely pulverized arsenic, mixed with about an ounce of fryed liver pudding, was given to a dog twelve months old. Immediately afterwards, a mixture of two ounces of hæmatite with seven ounces of liver pudding was offered to him, the whole of which he devoured, and then drank a small quantity of milk. Forty minutes afterwards, he was attacked with violent retching; the anterior extremities were extended spasmodically forwards, and the posterior drawn backwards, as is usual in cases of poisoning from arsenic; he also had severe vomiting, and convulsive spasms of the abdominal muscles. At eleven o'clock he went to his bed and was peevish; the retching continued during the afternoon, and he had five dark-coloured evacuations from the bowels. On the 17th of January, he remained the whole day in his bed, refused to eat or drink, and had six dark-coloured evacuations. On the 18th he appeared quite lively, sprang to meet the servant, and ate and drank with much desire.

*Experiment* 2. On the 19th of March, 1836, at eleven o'clock in the forenoon, a drachm of finely powdered arsenic, mixed with three-quarters of an ounce of liver pudding, was given to a dog four months old, and immediately afterwards an ounce and a half of rust of iron mixed with six ounces of liver pudding. The animal ate only three fourths of the antidote, and then drank a small quantity of milk. At half-past eleven he vomited, but still appeared lively; at one o'clock he had a green evacuation from the bowels. During the afternoon he had five alvine evacuations and frequent retching, but no vomiting. In the evening he went to his bed, and remained there quietly during the night; on the morning of the 20th, he had several dark brown fluid evacuations, but was in other respects lively, had a good appetite, ate bread, and did not exhibit the slightest trace of illness.

*Experiment* 3. On the 19th of March, 1836, at two o'clock in the afternoon, a drachm of finely powdered arsenic, which had been previously dissolved in two ounces of hot water, was poured down the throat of a dog four months old, while the solution was still warm, by means of a tin funnel, and about ten minutes afterwards, an ounce and a half of rust of iron, mixed with a quantity of milk, was administered in the same way. Five minutes afterwards, the dog was attacked with convulsions and retching, and dropt down as if dead. After fifteen minutes he got up, crawled into a corner, and vomited violently. During the night he had several dark-coloured alvine evacuations. On the 20th he was sullen, and refused to eat; on the 21st he was fresh and lively, and greedily ate some bread offered to him.

*Experiment* 4. On the 24th of March, 1836, at nine o'clock in the forenoon, a drachm of arsenic, mixed with an ounce of liver pudding, was given to a dog four months old, and then an ounce and a half of hæmatite, mixed with five ounces of liver pudding: the animal devoured the whole. Forty minutes afterwards, he was attacked with retching and convulsions followed by severe vomiting; as soon as the retching ceased, he ate up again what had been ejected from the stomach. At twelve o'clock he began to vomit again, became dejected, and went to his bed; he whined, retched continually, and his jaws were covered with a whitish foam. During the afternoon he had several alvine evacuations of a reddish colour, trembled, and was affected with constant retching and convulsive twitches. In the evening he was more tranquil. On the 25th he was quite lively, sprang to meet us, and ate with a good appetite.

*Experiment* 5. On the 24th of March, 1836, at eleven o'clock in the forenoon, a drachm of finely pulverized arsenic, mixed with an ounce of liver pudding, was given to a dog three months old, and immediately afterwards an ounce and a half of lapis hæmatites mixed with five ounces of liver pudding; the animal consumed only two thirds of the antidote. Twenty minutes afterwards he began to vomit, and at the same time had an evacuation from the bowels: he was then attacked with retching, vomiting, and convulsions, which lasted until one o'clock; during this time he vomited six times. At two o'clock he became dejected, shivered, and sought his bed; the retching was extremely severe, and his jaws were covered with foam. These symptoms disappeared about eight o'clock in the evening. On the 25th, he remained in his bed; about ten o'clock in the forenoon, he vomited twice a white frothy fluid; at eleven, he had some fluid evacuations from the bowels. On the 26th, he had several greenish brown evacuations, but appeared lively and ate with appetite.

From these experiments Dr. Von Specz is led to conclude, that rust of iron and hæmatite, although they do not prevent all the bad effects of arsenic on the system, may, in defect of the hydrated peroxyde of iron, be employed as antidotes to that poison. To the hydrated peroxyde, which is capable of neutralizing all the deleterious properties of the poison, he assigns the first rank as an antidote to arsenious acid, next to this stands rust of iron, and then, *sed longo intervallo*, hæmatite, which in consequence of its slow operation, may be used without any beneficial result where the poison is exercising a very powerful action on the system. Experiments 1, 4, and 5, of the second series, shew distinctly the predominant influence of arsenic on the system, although the hæmatite was administered immediately after the poison, and before its specific effects could be produced. The animals, it is true, did not die, but the counteracting powers of the antidote were not manifested until nearly three hours after its exhibition. These objections do not apply with any thing like the same force to rust of iron, which Dr. Von Specz thinks may be advantageously employed as an antidote in defect of the hydrated peroxyde. Its great efficacy as an antidote is shewn in Experiments 2 and 3 of the second series. The remarkable effects of the arsenic in Experiment 3 are to be attributed to the mode of administration, for Dr. Von Specz has repeatedly found that the poison operates much more rapidly when introduced into the stomach into a state of solution. A drachm of arsenic in powder does not produce its deadly effects on the system in less than six or eight hours, while the same quantity dissolved in warm water destroys life in a much shorter time. Rust of iron has also the additional advantage, that it can always be procured with facility.

*Med. Jahrbücher des k. k. O. St.* xix Band. 4 Stück. xx Band. 1 Stück. 1836.

---

*Case of Poisoning by Liquor Potassæ.* By DARTO-MASSART, Pharmacien at Mons.

MR. D., aged thirty-five, drank, instead of wine, a quantity of water of potash. Soon perceiving the error, he complained of severe pains in the epigastric region and nausea, and, in the course of a quarter of an hour, of general coldness; face pale, presenting the appearance of intense suffering. A solution of tartaric acid was administered, (four drachms to the pint of water,) and given at short intervals. Sinapisms were applied to the feet, and emollient fomentations to the abdomen, with frequent enemas. In a short time the symptoms abated, and he began to grow warm; a slight perspiration continued for two hours, followed by a black stool. Two days after, the tongue and back part of the mouth threw off a very thick and tough membrane. The patient took small quantities of broth, and shortly recovered his health.

*Gazette Médicale de Paris. November,* 1836.

